# Supplementary material for: Role of dynamical instability in QT interval variability and early afterdepolarization propensity
Source: Biophys J. Author manuscript; Available in PMC 2025 Oct 27. (PMC12414709; doi:10.1016/j.bpj.2025.07.017)
Supplement: Figures S1–S4 and supporting material [file NIHMS2117151-supplement-Figures_S1_S4_and_supporting_material.pdf]

Biophysical Journal, Volume *124*

Supplemental information

Role of dynamical instability in QT interval variability and early afterdepolarization propensity

Daisuke Sato, Bence Hegyi, Crystal M. Ripplinger, and Donald M. Bers

Supplemental Information

Role of Dynamical Instability in QT Interval Variability and Early Afterdepolarization Propensity

Daisuke Sato[*1], Bence Hegyi[*1], Crystal M Ripplinger[*1], Donald M Bers[*1]
[*1]Department of Pharmacology, University of California, Davis, California, 95616

Running title: Mechanistic link between QTV and EADs

Address for correspondence and Proofs:
Daisuke Sato, Ph.D.
Department of Pharmacology
University of California, Davis
Genome Building Rm 3503
Davis, CA 95616-8636
e-mail dsato@ucdavis.edu

v-f-x model of early afterdepolarizations

The minimal model for EADs is described by the following equations:

$$C_m \frac{dv}{dt} = -(I_{CaL} + I_K) = -(G_{Ca} d_\infty f (v - E_{Ca}) + G_K x (v - E_K)),$$
$$\frac{df}{dt} = \frac{f_\infty - f}{\tau_f},$$
$$\frac{dx}{dt} = \frac{x_\infty - x}{\tau_x} + \eta(t),$$

where C_m is the membrane capacitance, G_{Ca} is the maximum conductance of LTCC, d_∞ is the steady state of the activation of LTCC, f_∞ is the steady state of the inactivation of LTCC, E_{Ca} is the reversal potential of I_{CaL}, and G_K is the maximum conductance of the generic K current, E_K is the reversal potential of I_K, and τ_f is the time constant of the *f* gate. The variable *x* represents the activation of I_K. x_∞ is the steady state of *x* and τ_x is the time constant of *x*. $\eta(t)$ is the noise term, which satisfies the following correlation function:

$$\langle \eta(t)\eta(t') \rangle = \frac{\alpha_x(1-x) + \beta_x x}{N_x} \delta(t - t'),$$

where N_x is the number of channels, α_x is the opening rate, and β_x is the closing rate. The AP is initiated by a delta function that instantaneously shifts the voltage from the resting potential to +80 mV upon pacing. N_x=100,000 in this study; however, the results are qualitatively independent of N_x.
d_∞ and f_∞ are given by

$$d_\infty = \frac{1}{1 + \exp\left(-\frac{v+5}{6.24}\right)},$$

and

$$f_\infty = \frac{1}{1 + \exp\left(\frac{v-15}{8.6}\right)}.$$

Parameters

Parameter	Value
τ_f	50
G_{Ca}	5
G_K	10
E_{Ca}	60
E_K	-80
C_m	1

Figure S1

Detailed view of the data presented in Figure 2, focusing specifically on the PCL range of 500 ms to 800 ms. (A) APD (from Fig. 2A) for PCLs between 500 and 800 ms. Large variability is due to deterministic chaos. (B) APD (from Fig. 2C) for the same PCL range. Large variability is due to stochastic EADs. (C) APD variability (from Fig. 2D) for the same PCL range. Variability increases without EADs.

Figure S2

APD variability in rabbit ventricular myocytes. Data are from Ref. (38). In that study, variability was calculated as the short-term variability of APD, whereas in the present study, we quantify APD variability using the standard deviation of APD. Although we used the same dataset, the recalculated APD variability values are slightly different from the values reported in Ref. (38). (A) In healthy control rabbits, APD variability ranged from 3 to 7 ms. (B) In heart failure rabbits, it ranged from 3 to 13 ms.

Figure S3

APD variability for normal repolarization reserve AP model. Black: reduced repolarization reserve (same as Fig 2), Red: normal repolarization reserve. (A) APD vs. PCL. (B) APD variability vs. PCL.

Figure S4

Relationship between initial depolarization level and APD. (A) Variations in stimulation current strength lead to different initial membrane depolarization levels (B) Changes in the initial membrane depolarization level result in a biphasic APD response. (C) AP traces. Blue: I_{stim}= 35 μA/μF, Black: I_{stim}= 90 μA/μF, Red: I_{stim}= 52 μA/μF.

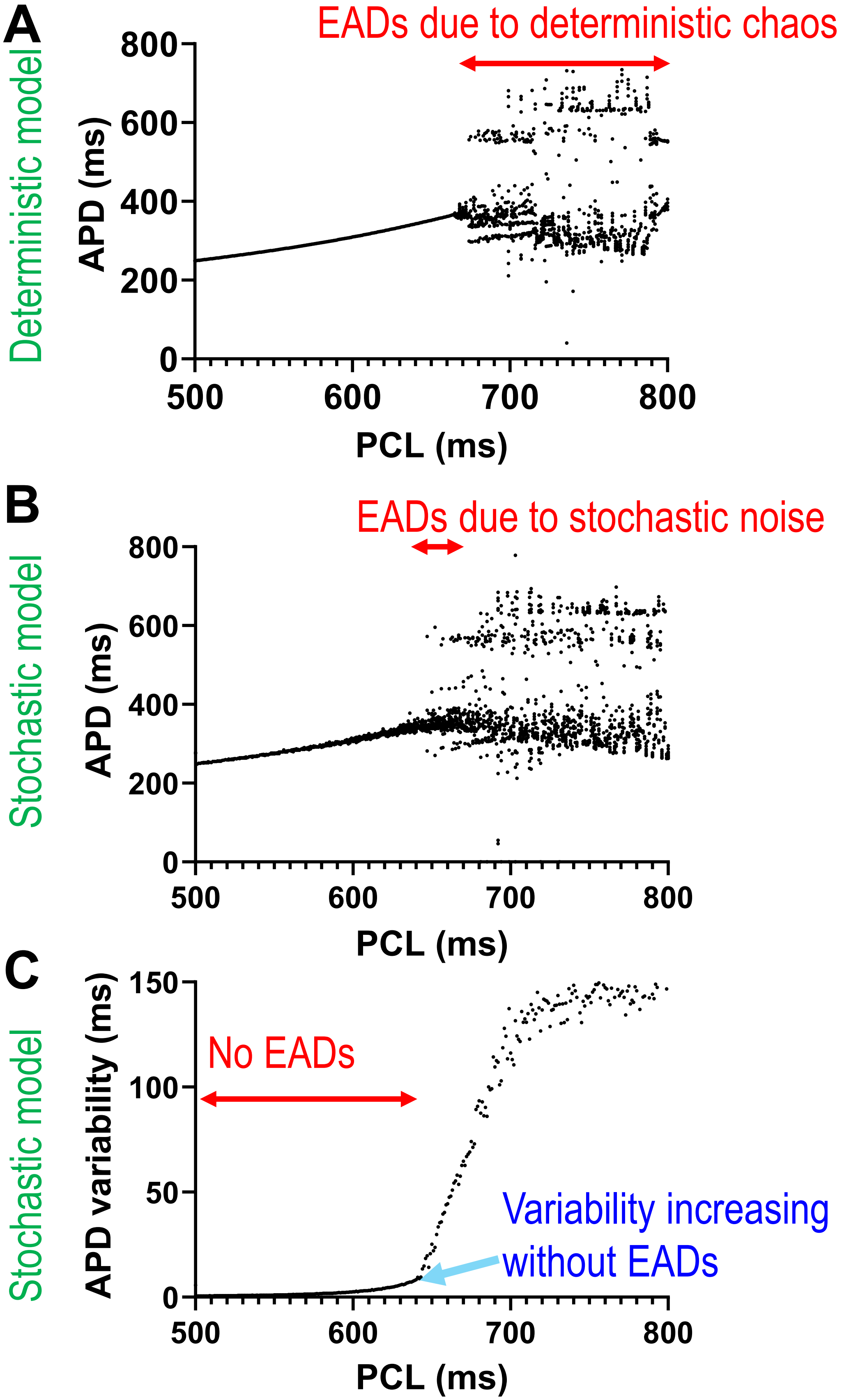
A
EADs due to deterministic chaos
Deterministic model
APD (ms)
800
600
400
200
0
500
600
700
800
PCL (ms)
B
EADs due to stochastic noise
Stochastic model
APD (ms)
800
600
400
200
0
500
600
700
800
PCL (ms)
C
Stochastic model
APD variability (ms)
150
100
50
0
No EADs
Variability increasing
without EADs
500
600
700
800
PCL (ms)

Figure S1

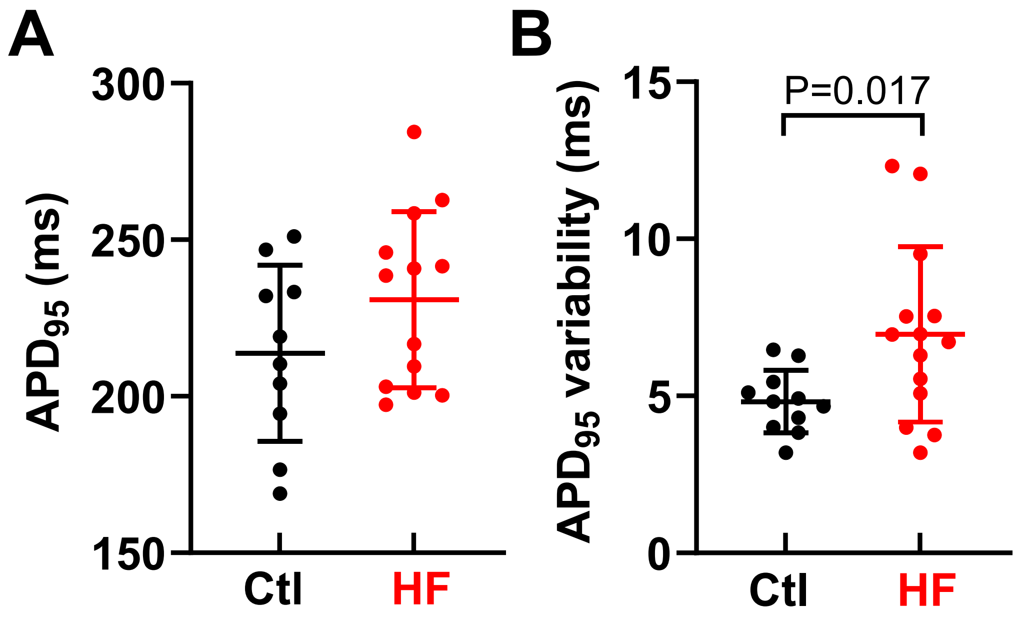
A
300
250
200
150
APD95 (ms)
Ctl
HF
B
15
10
5
0
APD95 variability (ms)
P=0.017
Ctl
HF

Figure S2

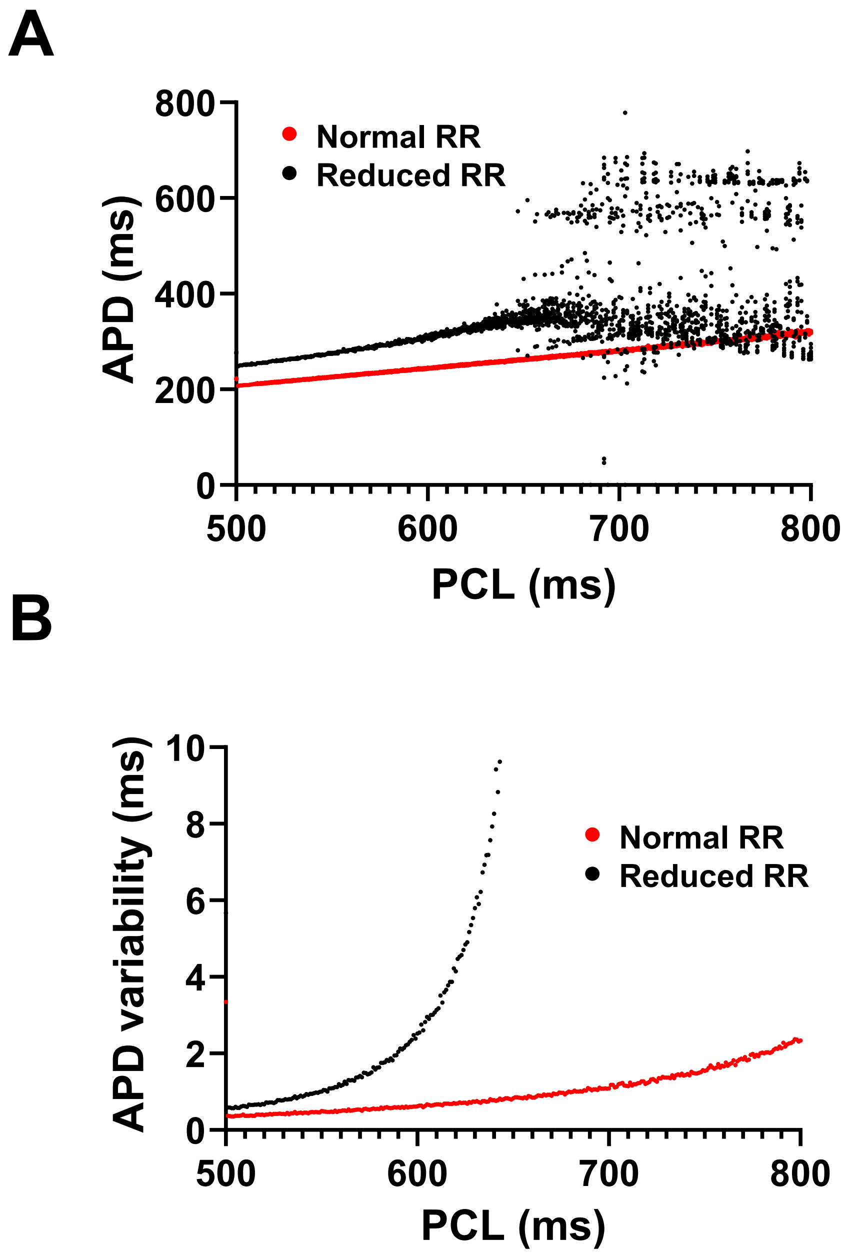
A
800
600
400
200
0
APD (ms)
Normal RR
Reduced RR
500
600
700
800
PCL (ms)
B
10
8
6
4
2
0
APD variability (ms)
Normal RR
Reduced RR
500
600
700
800
PCL (ms)

Figure S3

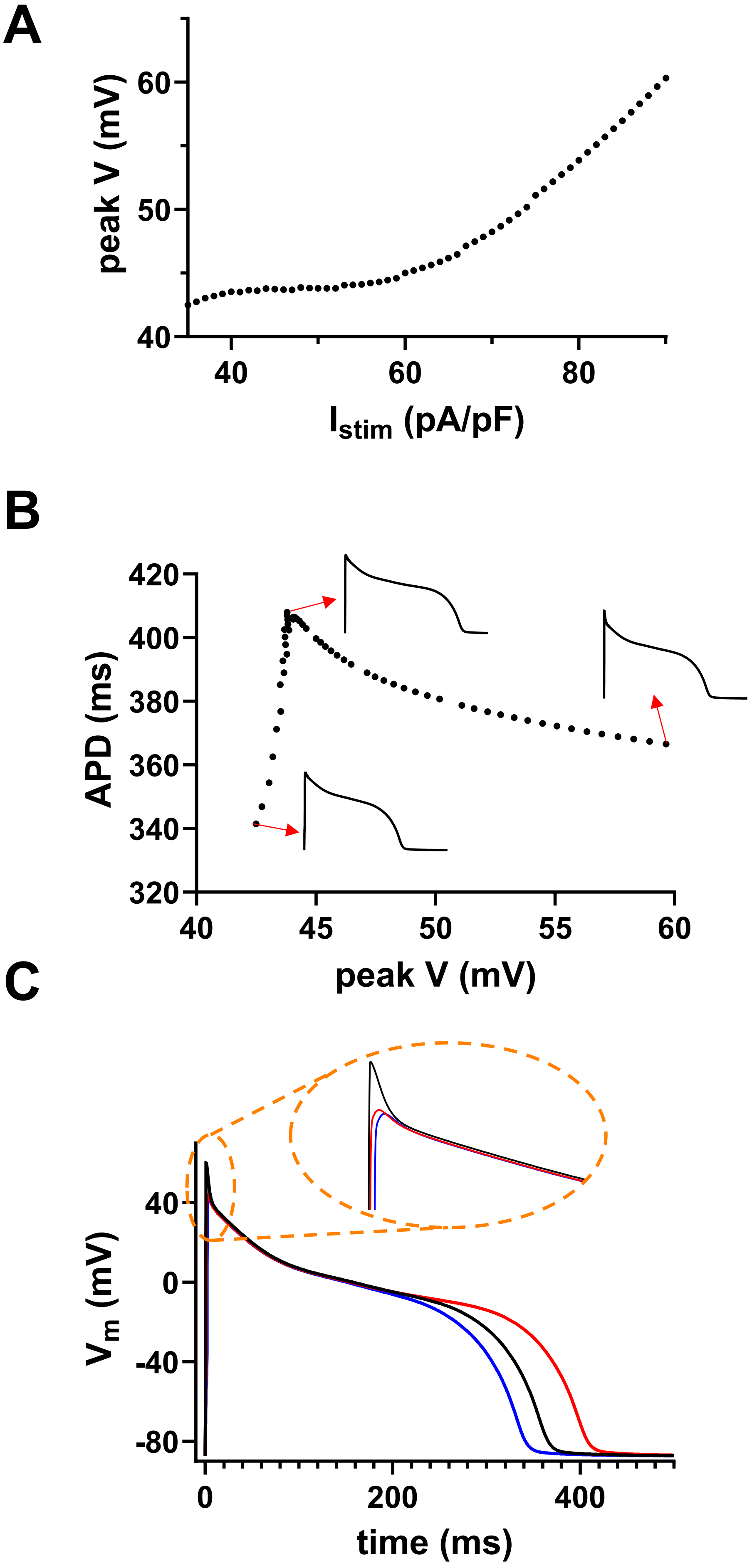
A
peak V (mV)
60
50
40
40
60
80
I_{stim} (pA/pF)
B
APD (ms)
420
400
380
360
340
320
40
45
50
55
60
peak V (mV)
C
V_m (mV)
40
0
-40
-80
0
200
400
time (ms)

Figure S4
